# Supplementary material for: Endometriosis and chronic pelvic pain have similar impact on women, but time to diagnosis is decreasing: an Australian survey
Source: Sci Rep. 2020 Oct 1;10:16253. doi: 10.1038/s41598-020-73389-2 (PMC7529759; doi:10.1038/s41598-020-73389-2)
Supplement: Supplementary file 1 — Supplementary Information. [file 41598_2020_73389_MOESM1_ESM.docx]

**Endometriosis And Chronic Pelvic Pain Have Similar Impact On Women, But Time To Diagnosis Is Decreasing: An Australian Survey**

Mike **Armour** ^1,2^*

m.armour@westernsydney.edu.au

Justin **Sinclair** ^1^J.Sinclair@westernsydney.edu.au

Cecilia H M **Ng**^3^cecilia.ng@unsw.edu.au

Mikayla S **Hyman**^4^mhyman124@gmail.com

Kenny **Lawson** ^2^K.Lawson@westernsydney.edu.au

Caroline A **Smith** ^1,2^caroline.smith@westernsydney.edu.au

Jason **Abbott** ^3^j.abbott@unsw.edu.au

^1^ NICM Health Research Institute, Western Sydney University, Penrith, NSW 2751, Australia

^2^ Translational Health Research Institute, Western Sydney University, Penrith, NSW 2751, Australia

^3^ School of Women's and Children's Health, University of New South Wales, Sydney, New South Wales, Australia

^4^ Department of Sociology/Anthropology, Middlebury College, Middlebury, VT, United States

*** Corresponding Author**

**Supplementary Table 1: Non-cyclical pelvic pain**

|  | **Endometriosis**  **(n=326 )** | **Chronic Pelvic Pain (n=64)** |
| --- | --- | --- |
| In the last 3 months, have you had pelvic pain at times **OTHER** than when you have your period or during/after intercourse? | 300 (92%) | 56 (87.5%) |
| **How long ago did this pain first start?^[[1]](#footnote-1)^** |  |  |
| *0-3 months* | 4 (1.4%) | 2 (3.6%) |
| *4-6 months* | 7 (2.4%) | 5 (8.9%) |
| *7-12 months* | 14 (4.8%) | 7 (12.5%) |
| *1-5 years* | 113 (39%) | 27 (48.2%) |
| *Over 5 years* | 152 (52.4%) | 15 (26.8%) |
| **Number of years pain first started (if more than 5 years)^[[2]](#footnote-2)^** | 12.6 (5.8) | 11.7 (4.7) |
| **Do you usually have this pain at about the same time in your cycle?^[[3]](#footnote-3)^** |  |  |
| *No* | 146 (50.3%) | 31 (55.4%) |
| *Yes – Mid cycle* | 92 (31.7%) | 19 (33.9%) |
| *Yes- before period* | 47 (16.2%) | 6 (10.7%) |
| *Yes- after period* | 5 (1.7%) | 0 |
| Approximately **how long in total** did you have this pain for in the **last 3 months?^[[4]](#footnote-4)^** |  |  |
| *Everyday* | 87 (30%) | 16 (28.6%) |
| *2-3 days/month* | 65 (22.4%) | 15 (26.8%) |
| *More than one day per week* | 106 (36.6%) | 17 (30.4%) |
| *Once per month* | 7 (2.4%) | 2 (3.6%) |
| *Less than one day/month* | 8 (2.8%) | 1 (1.8%) |
| *One day per week* | 17 (5.9%) | 5 (8.9%) |
| Do you take pain-killers for this pain, **prescribed for you by a doctor**?^[[5]](#footnote-5)^ | 152 (52.4%) | 16 (28.6%) |
| Do you take pain-killers for this pain **that you can buy without a prescription?** (e.g. Aspirin, Nurofen, Paracetamol)^[[6]](#footnote-6)^ | 240 (83.0%) | 46 (82.1%) |
| Please circle on the following scale, going from no pain (0) to worst possible pain (10), the number that indicates how severe your pain at times **OTHER than with periods or intercourse** has been ***on average*** in the **last 3 months^[[7]](#footnote-7)^** | 5.85 (1.9) | 5.36 (2.0) |
| Please circle on the following scale, going from no pain (0) to worst possible pain (10), the number that indicates how severe your pain at times **OTHER than with periods or intercourse** has been at its ***worst*** in the **last 3 months**. ^[[8]](#footnote-8)^ | 7.5 (2.0) | 7.0 (2.0) |

1. n_Endo_ =290, n_CPP_ = 56 [↑](#footnote-ref-1)
2. n_Endo_ = 107, n_CPP_ = 13 [↑](#footnote-ref-2)
3. n_Endo_ = 290, n_CPP_ = 56 [↑](#footnote-ref-3)
4. n_Endo_ = 290, n_CPP_ = 56 [↑](#footnote-ref-4)
5. n_Endo_ = 290, n_CPP_ = 56 [↑](#footnote-ref-5)
6. n_Endo_ = 289, n_CPP_ = 56 [↑](#footnote-ref-6)
7. n_Endo_ = 290, n_CPP_ = 56 [↑](#footnote-ref-7)
8. n_Endo_ = 287, n_CPP_ = 55 [↑](#footnote-ref-8)
